# Retraction Note: Temporal activation of anti- and pro-apoptotic factors in human gingival fibroblasts infected with the periodontal pathogen, Porphyromonas gingivalis: potential role of bacterial proteases in host signalling

**DOI:** 10.1186/s12866-019-1608-2

**Published:** 2019-10-17

**Authors:** Sonya Urnowey, Toshihiro Ansai, Vira Bitko, Koji Nakayama, Tadamichi Takehara, Sailen Barik

**Affiliations:** 1 Department of Biochemistry and Molecular Biology, University of South Alabama, College of Medicine, 307 University Blvd., Mobile, AL 36688-0002 USA; 2 Department of Preventive Dentistry, Kyushu Dental College, Kitakyushu, 803-8580 Japan; 3 Division of Microbiology and Oral Infection, Nagasaki University Graduate School of Biomedical Sciences, Sakamoto 1-7-1, Nagasaki, 852-8588 Japan

**Retraction note: BMC Microbiol 2006, 6**

**https://doi.org/10.1186/1471-2180-6-26**

The Editor has retracted this article [1] following an investigation by the University of South Alabama which found:

• The same Western blot images were used for Caspase-7 (Fig. 4C) and AKT (Ser473, MT + I) (Fig. 7)

• In Fig. 4D, the 6 h band for MT was replicated, the image was then inverted and the band was used for the 36 h time point

• In Fig. 7, the same Western blot image was used to demonstrate responses in MT + I for PDK-1 (P-Ser241) and P-GSK-3beta

• In Fig. 7, the blot for P-FKHR-L1 at time point 0 was replicated and used for the 24 h time point

Sailen Barik, Vira Bitko, Toshihiro Ansai and Sonya Urnowey do not agree with this retraction. Koji Nakayama and Tadamichi Takehara have not responded to any correspondence about this retraction.
